# MAGIC-SPP: a database-driven DNA sequence processing package with associated management tools

**DOI:** 10.1186/1471-2105-7-115

**Published:** 2006-03-07

**Authors:** Chun Liang, Feng Sun, Haiming Wang, Junfeng Qu, Robert M Freeman, Lee H Pratt, Marie-Michèle Cordonnier-Pratt

**Affiliations:** 1 Laboratory for Genomics and Bioinformatics, Department of Plant Biology, University of Georgia, Athens, GA 30602, USA; 2 Department of Computer Science, University of Georgia, Athens, GA 30602, USA; 3 Department of Systems Biology, Harvard Medical School, Boston, MA 02115, USA; 4 Department of Botany, Miami University, Oxford, OH 45056, USA; 5 Nanosphere, Inc., 4088 Commercial Avenue, Northbrook, IL 60062, USA; 6 Department of Genetics, University of Georgia, Athens, GA 30602, USA

## Abstract

**Background:**

Processing raw DNA sequence data is an especially challenging task for relatively small laboratories and core facilities that produce as many as 5000 or more DNA sequences per week from multiple projects in widely differing species. To meet this challenge, we have developed the flexible, scalable, and automated sequence processing package described here.

**Results:**

MAGIC-SPP is a DNA sequence processing package consisting of an Oracle 9i relational database, a Perl pipeline, and user interfaces implemented either as JavaServer Pages (JSP) or as a Java graphical user interface (GUI). The database not only serves as a data repository, but also controls processing of trace files. MAGIC-SPP includes an administrative interface, a laboratory information management system, and interfaces for exploring sequences, monitoring quality control, and troubleshooting problems related to sequencing activities. In the sequence trimming algorithm it employs new features designed to improve performance with respect to concerns such as concatenated linkers, identification of the expected start position of a vector insert, and extending the useful length of trimmed sequences by bridging short regions of low quality when the following high quality segment is sufficiently long to justify doing so.

**Conclusion:**

MAGIC-SPP has been designed to minimize human error, while simultaneously being robust, versatile, flexible and automated. It offers a unique combination of features that permit administration by a biologist with little or no informatics background. It is well suited to both individual research programs and core facilities.

## Background

**Figure 1:**
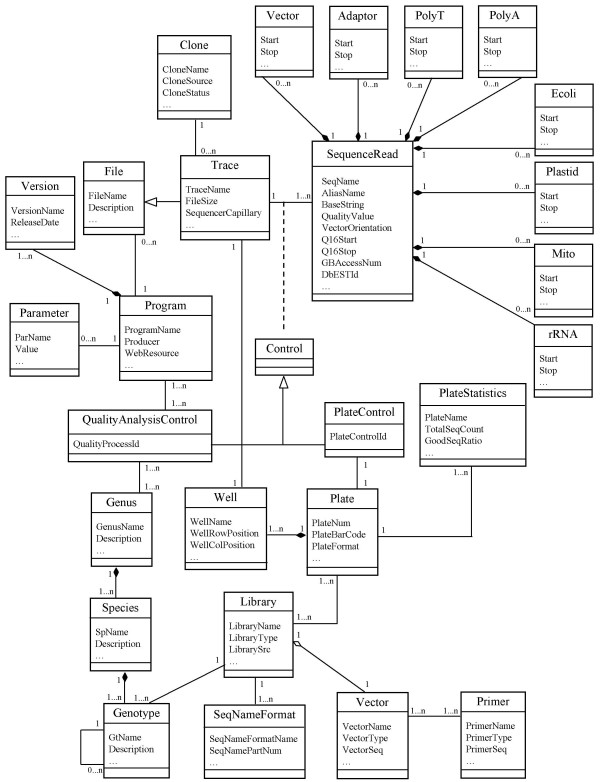
**Overview of UML class diagram for MAGIC-SPP database design**. Conventions used in the presentation of this diagram are as defined by Favre [18].

As an individual research laboratory we began high-throughput DNA sequencing in 1998. Accumulated experience with five independent expressed sequence tag (EST) projects (sorghum, pine, horse, human, and the parasitic ciliate *Ichthyophthirius*), BAC shotgun sequencing, and a variety of *ad hoc *applications, including processing of sequences produced by other laboratories or sequencing centers, led to a system redesign in 2002. The result was the creation of a **m**odular **a**pproach to a **g**enomic, **i**ntegrated and **c**omprehensive **d**ata**b**ase (MAGIC DB) and its interfaces, which is designed for gene discovery and expression applications [1]. MAGIC-SPP, which is the focus of this contribution, was created to meet our need for a database-driven sequence processing package, many parameters of which could be controlled by biologists using a web-based graphical user interface (GUI). While MAGIC-SPP can function as an independent tool, maximum value is realized when it is used as an integral part of the complete MAGIC system (Fig. 1 in Additional file 1).

Arguably the most widely used independent tool for sequence processing today is Lucy [2], which has become part of the data processing pipelines of several genome sequencing centers [3-5]. ESTprep is a more recent independent tool for pre-processing raw cDNA sequence reads, identifying features such as cloning vector and restriction sites, and assuring that only high-quality reads proceed to later stages of data analysis [6]. Sequence processing tools have also been developed as part of more comprehensive packages, including the Staden Package [7], MTT [8], Hopper [9], Gasp [10], and MGI [11]. These packages typically include additional functionalities such as clustering and annotation. Recently, packages have also been developed specifically for working with ESTs. PipeOnline is an automated pipeline that transforms large collections of raw cDNA sequences into a unigene EST data set [12]. PartiGene is a generic and automated pipeline for processing raw trace files into a partial genome database [13]. ESTAP is a set of analytical procedures for verifying, cleaning and analyzing ESTs [14], while ESTWeb is an internet-based package designed for processing and storing EST data [15]. All use a relational database to store the processed data, having adopted different methods for processing raw sequences. These packages individually, however, do not provide a comprehensive package of platform independent GUIs that a biologist with few or no informatics skills requires to fully manage a diversity of DNA sequencing projects, from creation, through quality control in the laboratory, to completion.

MAGIC-SPP has been developed in part to fill this latter need and in part to create an improved sequence processing pipeline. Thus, it provides not only a flexible and automated sequence processing pipeline controlled by a relational database, but also a laboratory information management system (LIMS) and a comprehensive set of interfaces to administer sequencing projects, explore sequences, monitor quality control, and troubleshoot problems in the laboratory. It consists of an Oracle 9i database, a Perl processing pipeline, and interfaces implemented with either JavaServer Pages (JSP) or a Java GUI. MAGIC-SPP is robust, having already processed more than 825,000 sequences into MAGIC DB, of which 210,000 were produced by four other sequencing facilities. These sequences have been processed for 174 independently defined projects, each typically consisting of a BAC or a cDNA library. Its implementation at The University of Georgia currently serves 112 user accounts organized into 15 access groups at a total of 11 universities or government laboratories. The package has also been installed at three additional sites. When MAGIC-SPP is used in conjunction with the comprehensive MAGIC DB system, it is integrated with tools for clustering ESTs, for mining and visualizing the results of such clustering, for electronic annotation of sequences, and for integration with MIAME-compliant microarray data [1]. It is ideally suited for use by both individual research programs and core facilities, possessing a combination of features presently unavailable in any other single package. Here we describe the schema and sequence processing pipeline of MAGIC-SPP, together with an introduction to the interfaces that are part of this package. About 500,000 sequence reads produced with MAGIC-SPP can be explored at and downloaded from a publicly accessible implementation of MAGIC DB using MAGIC SeqView [16].

## Implementation

### Database design

**Figure 2:**
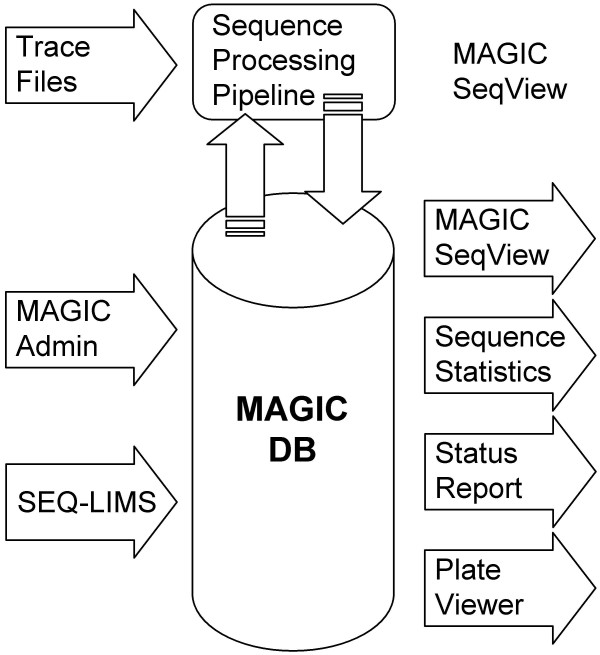
**Overview of the components of MAGIC-SPP**. MAGIC Admin facilitates administration of MAGIC DB, while MAGIC SEQ-LIMS can be used optionally to track all sequencing activities in the laboratory. Using information retrieved from MAGIC DB, the sequence processing pipeline calls bases, trims and cleans sequences, and updates the database. Three data mining/visualization interfaces, Sequence Statistics, Status Report, and Plate Viewer, permit biologists to monitor progress and quality control and to trouble shoot problems in the laboratory. Sample screen shots of these interfaces are presented in Additional file 1. MAGIC SeqView is a Java GUI that facilitates exploration of sequences and downloading of selected sequences in fasta format.

An Entity-Relationship data model [17] was used in the conceptual database design phase. The complete Entity-Relationship diagram for that part of MAGIC DB utilized by MAGIC-SPP is presented in Figure 2 of Additional file 1. Figure 1 provides the MAGIC-SPP class diagram in Unified Modelling Language (UML)[18]. Because it was determined that the database should not only serve as a biologist's notebook and a repository of processed data, but should also support automated, flexible and intelligent software for processing DNA sequences, many data objects in MAGIC-SPP are used to address the following issues: how to process, what to process and where to store information.

As shown in Figure 1, **Clone **is the class representing the DNA to be sequenced, while **Trac**e is a class describing trace files derived from sequencing DNA clones. **Clone **has a one-to-many relationship with **Trace**, permitting multiple sequences to be obtained from the same target DNA. Trace files are processed by phred [19] to create sequence reads, characterized by the class **SequenceRead**. The MAGIC sequence processing pipeline focuses on creation of one or more sequence reads from each trace file and identifies the locations of regions of good quality, vector and adapter fragments, poly-T and poly-A tails, and contaminating DNA.

Two classes, **QualityAnalysisControl **and **PlateControl**, deal with the issue of "how to process" (Fig. 1). **QualityAnalysisControl **defines high-level control of processing, such as organism-specific processing, whereas **PlateControl **defines low-level processing, such as processing trace files for a particular plate or wells within a plate. The processing pipeline needs to know how many procedures or steps are required, which program and program version will be used for each procedure or step, which parameters should be applied, where the associated executable and input files are located, and so on. That is why **QualityAnalysisContro**l has been associated with, among others, the classes **Program**, **Version**, **File **and **Parameter**. Sequence quality analysis is also organism specific. For example, human sequences do not need to be screened for chloroplast DNA whereas sorghum sequences do. Similarly, sorghum sequences should be screened against its own mitochondrial genome sequence, not that of human. Therefore, **Genus **and **Species **are associated with **QualityAnalysisControl **as shown in Figure 1. As described further below, a key feature of **QualityAnalysisControl **is that each *QualityProcessId *(italics will be used to denote a field or column in a table) defines a specific workflow or protocol for processing raw trace files. Any change in any part of the sequence processing pipeline, such as a change in phred version or modification in parameters for vector/adapter screening, results in a new *QualityProcessId*.

**PlateControl **relates to both the "how to process" and "what to process" questions. Each trace file must be associated with a specific well in a defined 96- or 384-well sequencing plate. In high throughput DNA sequencing, plates are usually homogeneous with respect to template source, vector orientation, and primer usage. A single plate, however, can sometimes be heterogeneous with respect to these parameters. This latter situation is handled by the complex associations among **Plate**, **Library**, **Vector **and **Primer **classes as shown in Figure 1. The key feature of **PlateControl **is that it serves as a bridge to bring to a processing pipeline all low-level information about processing, such as the type of sequencing performed (EST, shotgun, primer walking, etc.), the identity and type of library sequenced, the vector and primer used, the direction of a sequence with respect to vector and, if dealing with a cDNA library, the associated EST direction. This bridge facilitates accurate processing of individual trace files obtained from a single plate containing samples that are heterogeneous as well as homogeneous with respect to parameters such as those enumerated here.

It is often important to associate sequence reads with genotype. Moreover, libraries to be sequenced are frequently prepared from a mix of two or more genotypes. Thus, **Genotype **is a self-associated class that is also associated with **Library **(Fig. 1). MAGIC-SPP utilizes *GtCombineCode *to define different genotype associations (not shown in Fig. 1). Embedding this *GtCombineCode *into sequence names has proven to be extremely useful for downstream data mining and visualization of features such as sequence polymorphisms, for which genotype information is critical. We are unaware of any other processing pipeline that includes this genotype-association feature as an integral component of the sequence name.

**Library **has a one-to-many relationship with **SeqNameFormat**, which supports different conventions for naming trace files and resultant sequence reads. **SeqNameFormat **permits processing of trace files using any naming convention as long as the name is clearly defined in terms of delimiter and/or component positions. An example of the MAGIC naming convention is EM1_17_A03.b1_A002, in which (_) and (.) are delimiters. Essential components include project or library name (EM1), plate number (17), well identification (A03), sequence direction relative to the vector (b), a numeral (1) that is incremented each time a new trace file is obtained from the same clone with the same sequencing primer, and the appropriate *GtCombineCode *(A002), as described in the preceding paragraph. Among them, a project or library name, plate number, well identification and sequence direction relative to vector are required to be present in the name of a trace file if it was named by any convention other than ours. When processing such an external trace file, all of these essential components can be obtained by parsing the trace file name and/or retrieving from the MAGIC database information entered into it by means of user interfaces, if that information is not present in the trace file name. Using the class **SeqNameFormat**, a new name in our convention is created as an external trace file is processed. Consequently, for an external trace file a sequence read will then have two names coexisting in the database such that it can be accessed by either. In this way, all trace files processed into MAGIC DB by this pipeline can profit from downstream data mining/visualization tools without requiring a user to work with the MAGIC naming convention.

Many classes are used for storing processed data. As already noted, **SequenceRead **describes sequence reads, including base calls and their associated phred quality scores. **SequenceRead **describes the results from processing trace files using a specific workflow or protocol defined in **QualityAnalysisControl**. **SequenceRead **is also an aggregate class because it has a "whole-part" relationship with many other classes, including **Vector**, **Adapter**, **PolyT**, **PolyA**, and so on. The table for **SequenceRead **has a primary key of *QualityProcessId *and *SeqName *(not shown in Fig. 1), which is inherited as foreign keys in the tables for its component classes. This design allows sequence reads and associated information derived from different implementations of the sequence processing pipeline to coexist in the database, thereby facilitating comparisons among different sequence processing parameters and/or components, such as different versions of phred.

**SequenceRead **and its component classes are designed to represent sequence features at the level of individual trace files. During a sequencing project, biologists need to monitor progress and quality control, and to troubleshoot problems in the laboratory in a timely fashion. **PlateStatistics **was created to assist with these activities via the **PlateControl **class. It hosts plate-level information about sequence processing, which has a many-to-one relation to **Plate**.

### Software design

The core of MAGIC-SPP is the processing pipeline, which processes trace files in stepwise fashion as described in the next section. Foundation Classes serve as a layer of abstraction to the relational database by encapsulating various SQL queries for retrieving information from the database and for updating the database as processing proceeds. Because some third-party software such as phred [19], CROSS_MATCH [20] and SSAHA [21] are used, Wrapper Classes have been created to execute the third-party software with the capability of adjusting parameters and parsing results. Processing Classes mediate between Foundation and Wrapper Classes to accomplish a given processing step or procedure.

Because the database not only serves as a data repository but also controls processing, several user interfaces have been developed to support and/or take advantage of these dual functions. Figure 2 illustrates the relationships among the sequence processing pipeline, MAGIC DB, and the various interfaces that provide input to the database and facilitate typical database queries of interest to the biologists who use MAGIC-SPP. The relationship between MAGIC-SPP and the entire MAGIC system is illustrated in Figure 1 of Additional file 1.

**Figure 3:**
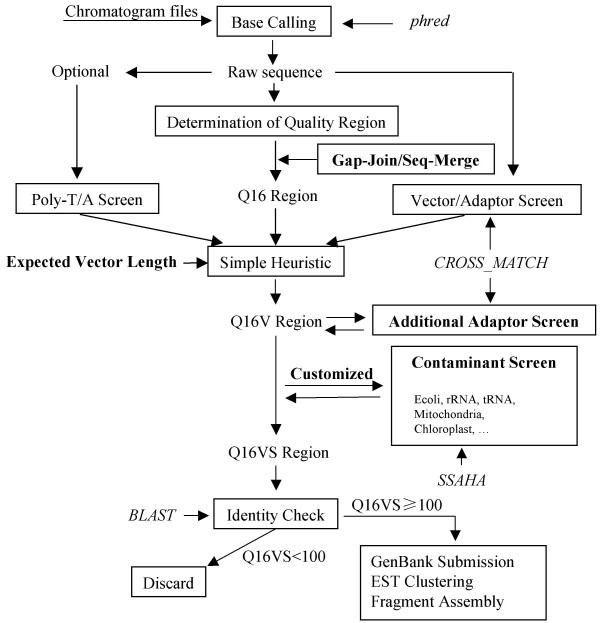
**Dataflow for the sequence processing pipeline of MAGIC-SPP**. The Q16 Region is defined using four moving windows in conjunction with Gap-Join/Seq-Merge. The Q16V Region is the Q16 Region trimmed of vector and adapter, while the Q16VS Region is the Q16V Region screened for contaminating sequences. Novel features of this pipeline are in bold font.

MAGIC Admin is a GUI designed to facilitate for a biologist the entry of information required for control of processing. This information includes genus, species, genotype, vector, primer, colony picking, sequencing targets, and so on (see Fig. 3 in Additional file 1). It is accessible only to users who have been granted administrative privileges for a given sequencing project. Among other functions, this interface is also used to create access groups and user accounts, to control read and write access to projects by access group or by individual user account, and to enter information required for GenBank depositions.

**Figure 4:**
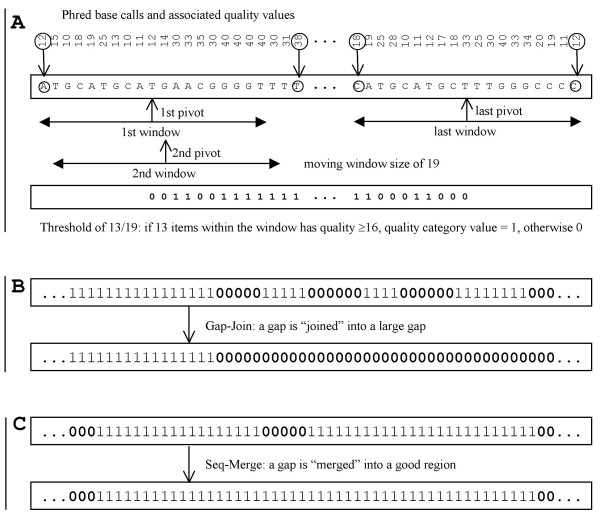
**Moving windows categorize each base call as bad (0) or good (1)**. Illustrated here is the function of moving_window (19,10,13/19), the last of four such moving windows. (A) Each base call and its associated quality score is determined with phred. A moving window categorizes each pivot base as either bad or good, resulting in a new quality category string consisting of 0 s or 1 s. Gap-Join/Seq-Merge evaluates gaps identified by two of the moving windows as described in the text. This algorithm then either joins two gaps to form a longer one (B) or, when the next fragment of good quality is at least twice as long as the preceding gap length, merges that gap into an extended good region (C).

MAGIC SEQ-LIMS automates and tracks sequencing activities, from picking bacterial colonies, through thermal cycling, to automatic database-directed creation of plate records for an ABI 3700 or 3730 sequencer (see Fig. 4 in Additional file 1). It serves as a notebook for technicians as well as a tool to minimize human error and ensure that standard operating procedures are followed. MAGIC SEQ-LIMS is not, however, required to utilize MAGIC-SPP. We have, for example, used MAGIC-SPP to process into MAGIC DB ~210,000 trace files produced by other sequencing centers. As already noted, the sequence reads could in these cases be accessed by both the original naming convention and the standard convention used by MAGIC.

**Figure 5:**
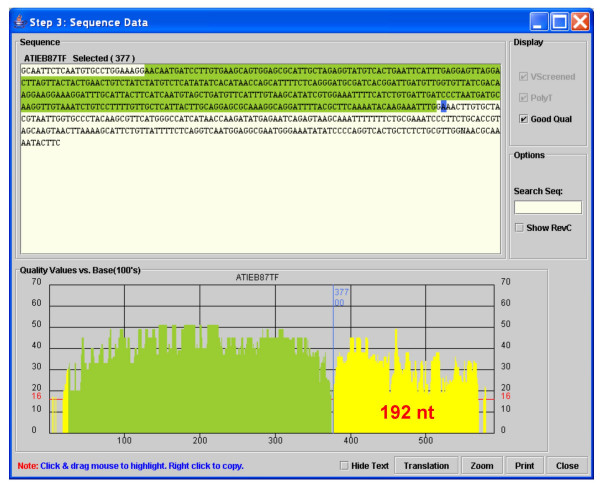
**Screen shot of a sequence read obtained with Lucy [2]**. The sequence read is from a demonstration data set [27]. It is displayed with a version of MAGIG SeqView [1] modified to display sequence reads trimmed by Lucy. The upper window displays base calls; the lower provides phred quality scores in a histogram, with the first base call at the left. The quality-trimmed region provided by default parameters is green. The gap centered at nucleotide 377 is identified by the base call in blue in the upper window and the vertical blue line in the lower window. This gap contains three base calls with phred quality scores of zero, each of which yields an error probability of 1. The average error probability for a window of 10 is 0.304, exceeding the default maximum of 0.3 for _window_size2. Consequently, Lucy divides the original high quality region into two segments, choosing the longer of the two as the final result. Because the length of the shorter high quality region is sufficiently long, Gap-Join/Seq-Merge in MAGIC-SPP would join these two regions, thereby increasing the content of high quality base calls by 192 nucleotides.

**Figure 6:**
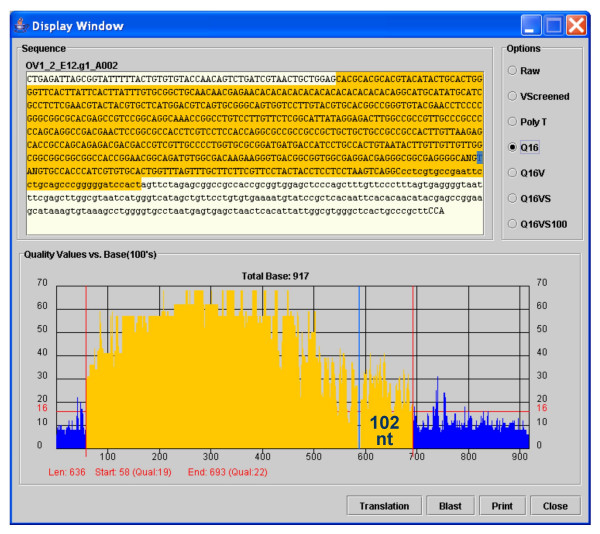
**Screen shot of a sequence read obtained with MAGIC-SPP**. The sequence read was obtained from a publicly accessible implementation of MAGIC DB and is displayed with the MAGIC SeqView GUI available at that web site [22]. The upper window displays base calls; the lower provides phred quality scores in a histogram, with the first base call at the left. The quality-trimmed region provided by default parameters is in orange. The center of the gap at nucleotide 588 is highlighted in blue in the upper window and marked by a vertical blue line in the lower window. In a window of 10, the average error probability of this gap is 0.402, sufficient for Lucy to divide the high-quality region into two segments when used with default parameters [2]. Lucy would then pick the longest segment as its final output. In contrast, the length of the right-hand high quality segment is sufficient for the Gap-Join/Seq-Merge algorithm when used with default parameters to merge the two regions, providing an additional 102 high quality base calls in the final quality-trimmed output.

A third interface, Sequence Statistics, displays information about sequencing yield and quality for selected plates and/or for an entire project. For an example of the information provided, see Figure 5 in Additional file 1. Two additional interfaces, Status Report and Plate Viewer, monitor progress during the course of a project and provide an aid for troubleshooting problems in the sequencing laboratory. For examples of these two interfaces, see Figures 6 and 7 in Additional file 1.

A Java GUI, MAGIC SeqView, displays both base calls and their individual phred quality scores [1]. It displays sequence reads by both their original name and that used by MAGIC, as well as considerable other information about each sequence read. Sequence reads can be trimmed in several ways and can be downloaded from the database in fasta format as trimmed. They can also be translated, viewed and downloaded in all six reading frames. This Java GUI can be explored in conjunction with a public implementation of MAGIC DB [16].

### Sequence processing pipeline

An overview of the sequence processing pipeline is presented in Figure 3. New features here include introduction of a Gap-Join/Seq-Merge algorithm for extending the useful length of sequence reads, the concept of Expected Vector Length, an additional adapter screen to identify instances of adapter concatenation, and automated organism-specific screening for contaminants facilitated by the database and MAGIC Admin.

#### 1. Determination of quality region – Gap-Join/Seq-Merge

Inputs for this step are conventional fasta sequence and quality files. Four types of moving windows, each with a step size of 1, categorize each base call as either bad (0) or good (1), as illustrated in Figure 4A for the fourth and final moving window. Each of these four windows examines the array of phred quality scores, using 16 as the threshold for a base call to be considered passing. The first is moving_window (100,1,80/100), with a size of 100, pivot start position of 1, and threshold of 80/100. In this window the pivot start position is the first called base, although for the subsequent three windows it is in the middle. If 80 of the 100 base calls in this moving window have a quality score of 16 or greater, then the pivot base is categorized as good. If no base calls are categorized as good, then the sequence will not be examined further. Sequences that pass this first window are subsequently examined by two additional windows of varying length to identify short regions of low quality called gaps. Gaps between 5 and 9 nucleotides long are identified by moving_window (9,5,5/9), while those at least 10 nucleotides long are identified by moving_window (19,10,10/19). All gap information is recorded in the database.

The fourth and final window is moving_window (19,10,13/19), which is used to determine the final high quality region (Fig. 4). It has a more stringent threshold for categorizing a base call as good than does the moving window that identifies long gaps (13/19 in place of 10/19). Gap-Join/Seq-Merge determines whether or not an internal gap or gaps will be included in the final high-quality region. Within the new array of quality category (0 or 1), the first and last fragments of high quality that have a continuous string of at least 20 good base calls (1 s) are identified. Starting from this first fragment and moving towards the last along the quality category array, each gap is examined with reference to its immediately following high-quality fragment. If the length of this high-quality fragment is at least twice the length of the gap, then the gap is merged with its two adjacent high-quality fragments into a longer fragment of high quality (Fig. 4C). Otherwise, the gap is joined with one or more adjacent gaps into a larger gap as shown in Figure 4B. This procedure is conducted iteratively until the last fragment is finished. In the end, only one high-quality region, called the Q16 Region, will remain. It is defined by *Q16Start *and *Q16Stop *in the table for **SequenceRead**. This region could be only the first high quality fragment, or it could be the result of merging this fragment with one or more gaps and other high quality fragments.

#### 2. Vector/adapter screening

Initial screening is done with the appropriate vector/adapter fasta file, the name and location of which is retrieved from the database. Vector and adapter fragment(s) are identified with CROSS_MATCH [20]. Simple heuristics are used to categorize a fragment as either VF1 (Vector Fragment 1, including any adapter; beginning of sequence read) or VF2 (Vector Fragment 2, also including any adapter; following the insert, if the latter is short and if the read length is sufficiently long). The concept of Expected Vector Length, which is the expected length from the last nucleotide of the sequencing primer to the first nucleotide of the insert for a given library when sequenced with a given primer, is an important component of these heuristics.

The Expected Vector Length is defined as the sum of the following two parts:

(1) The number of nucleotides from the end of the primer, but not including any nucleotide that is part of the primer, to the last nucleotide immediately prior to the sequence recognized by the restriction enzyme in the multiple cloning site closest to the primer. This number does not include any nucleotide that is part of the restriction enzyme recognition sequence.

(2) The number of nucleotides from the first nucleotide at the beginning of the first restriction enzyme in the multiple cloning site, which is the same enzyme as in (1) above, to the last nucleotide prior to the beginning of the insert. This last nucleotide will be one of the following: (2.1) the last nucleotide of the adapter, if one is used or (2.2) the last nucleotide of the strand that contains the primer sequence after the vector has been cut by the cloning enzyme.

Both values are entered into the database with MAGIC Admin. By defining the values in this fashion, maximum flexibility is achieved with respect to different primer-vector combinations and different cloning strategies with the same vector.

If a sequence has a vector segment with a start position less than the Expected Vector Length plus an allowance for sequencing error, then it is categorized as VF1. Otherwise it is categorized as VF2, unless its offset is less than the offset of the Q16 Region. If the total length of vector fragments within the Q16 Region is at least 90% of that region, then the sequence is tagged as total vector (Vtot). This threshold of 90% is user adjustable. A sequence tagged as Vtot is set aside, whereas other sequences are subject to further processing. The high-quality region after masking for vector and adapter is termed the Q16V Region. For some sequences, this region is not completely free of vector and adapter due to low quality base calls and occasional concatenation of adapters during library construction. To address this potential problem, an additional adapter-screening step is performed on the Q16V Region.

#### 3. Additional adapter screening

This additional screening also uses CROSS_MATCH [20], but applies more stringent parameters. Screening for an adapter sequence is done only in the expected location as defined by Expected Vector Length. For this step an adapter fasta file consists of up to two adapter sequences, one for each end of an insert. The minimum requirement for CROSS-MATCH to work properly is an adapter length of eight.

#### 4. Poly-A/T screening

When requested through MAGIC Admin, MAGIC-SPP can include this optional step. Two major phases are involved: detection of all poly-A/T segments and identification of one that is valid. If desired, the identified poly-A/T segment can be removed from the Q16V Region. Because poly-A and poly-T screening use a similar algorithm, we describe here only the latter. In the detection phase, all segments with a specified length of continuous T are detected. For each segment, an internal error for a specified number of bases is allowed. The default value for length is 10 with internal error of 2. In the identification phase, MAGIC-SPP first tries to merge in sequential order individual poly-T segments using a defined criterion, starting from the beginning of each sequence. The criterion involves evaluating the phred quality scores for bases in each gap between two adjacent poly-T segments and comparing the sizes of gaps and poly-T segments. A simple heuristic identifies a valid poly-T tail after merging segments. If VF1 is detected, then to be valid a poly-T must follow VF1 immediately. If VF1 is not detected, then for a poly-T to be valid it must be within the Expected Vector Length plus 20 nucleotides from the start of the sequence read. The value of 20 is user adjustable.

#### 5. Contaminant screening

Contaminants might arise from many sources, including *Escherichia coli*, mitochondria, chloroplasts and rRNA. Contaminant screening, which is controlled by the database, is organism specific. The processing pipeline executes different screening programs intelligently, using for different organisms and projects the predefined information entered into the database with MAGIC Admin. In addition, a user can easily turn on/off the optional screening modules that are organism or project specific. Significant matches with potential contaminant sequences are identified with SSAHA [21]. Relevant sequences are tagged with reference to the source of contamination. When a sequence is tagged as rRNA or as an *E. coli *contaminant, it is excluded from further processing. Sequences identified as mitochondrial or chloroplast contaminants are tagged as such, but nonetheless optionally proceed to further processing. That part of a sequence read that is screened for contaminants is referred to as a Q16VS Region.

### System implementation

MAGIC-SPP utilizes three servers: database, web and analysis. The production database is implemented with Oracle 9i in a Dell PowerEdge 6400 (4 × 700 MHz processor, 8 GB RAM, 200 GB disk space) using Windows 2000. A publicly accessible database [22], as well as databases established for developmental and educational functions, are in Dell Optiplexes using RedHat Linux AS3.0. The web server uses Tomcat version 4.27 on a Dell workstation (1 × 2.0 GHz processor, 512 MB RAM, 40 GB disk space). The analysis server is a second Dell PowerEdge 6400 (4 × 700 MHz processor, 8 GB RAM, 1300 GB disk space) using RedHat Linux AS3.0. All three components have been bundled successfully in a single desktop machine with RedHat Linux version 7.1 and above (1 × 2.0 GHz processor, 1 GB RAM, 100 GB disk space).

The processing pipeline has been developed with Perl 5.8.0. User interfaces MAGIC Admin, Sequence Statistics, Status Report and Plate Viewer are implemented with JSP. MAGIC SEQ-LIMS is also implemented with JSP, but XML-RPC (Remote Procedure Calls) is used to make remote procedure calls within JSP. MAGIC SeqView is implemented as a stand-alone Java GUI delivered by Java Web Start. All of these client user interfaces will run on RedHat Linux, Mac X, and Windows operating systems.

## Results and discussion

MAGIC-SPP is designed to be a database-driven DNA processing package, in which the relational database not only serves as a data repository, but also controls processing. The extensive interaction of MAGIC-SPP with a database to control processing permits a biologist with little or no informatics background to administer sequencing projects using the interfaces provided with it (Figs. 3, 4, 5, 6, 7 in Additional file 1). Substantial effort has also been invested in addressing the issue of database performance and extensibility during the design phase. For example, the primary key (*QualityProcessId*, *SeqName*) in the table for the aggregate class **SequenceRead **is inherited as foreign keys in tables for its component classes. One advantage of this design is that it facilitates greater data normalization and minimization of linking tables. A similar design has been adopted independently by The *Arabidopsis *Information Resource to improve database performance [23]. In addition, improvements have been made to the typical sequence processing pipeline. In this section, we will emphasize those features that distinguish MAGIC-SPP from other available options.

In recent years automated and flexible workflow designs have been developed for bioinformatics analyses downstream from sequence processing [24-26]. The inclusion of an administrative interface designed for use by a biologist, combined with database control of sequence processing, provides MAGIC-SPP with a comparable capability for processing DNA sequences. For example, once a biologist has inserted into the database the appropriate information about a project, organism-specific screening is fully automated. Similarly, if the SEQ-LIMS interface is used, then plate records for the sequencer are automatically produced by the database, thereby minimizing human error and ensuring that trace file and sequence read names are created correctly. Moreover, inclusion of **PlateControl **and its associated classes extends this biologist-directed automation down to the level of individual wells in a plate, providing maximum flexibility in application of the sequence processing pipeline. The inclusion of **SeqNameFormat **ensures that MAGIC-SPP can also be used in similarly automated fashion with naming conventions other than its own, yielding sequence reads that can be identified by either convention. In addition, by letting a biologist specify the goals of a project, such as the total number of sequences to be obtained in each direction, the Status Report interface (Fig. 6 in Additional file 1) automatically reports progress upon request for any selected time interval.

The inclusion of *QualityProcessId *in **QualityAnalysisControl **provides, of course, a way to associate with each sequence read a wealth of information about how it was processed or obtained from a trace file. In addition, however, the combined use of *QualityProcessId *and *SeqName *as primary key in the table for **SequenceRead **facilitates reprocessing of a trace file without compromising database integrity. Consequently, a biologist can directly explore in high throughput fashion different processing parameters, methods, and/or program versions. It becomes a trivial exercise to compare even tens of thousands of sequences processed by both new and old versions of phred to determine whether it is appropriate to switch to the new version.

Accurate identification of vector and adapter fragments can sometimes be very difficult, especially because regions of low quality typically occur at the ends of sequences where these fragments are typically found. To address this problem Lucy has been modified to handle special and/or difficult cases [2]. Occasionally, this approach results in reciprocal conflicts that Lucy then attempts to mediate. MAGIC-SPP deals with this problem, at least at the beginning of sequences where it is most severe, by instead introducing the concept of Expected Vector Length. As defined in the Vector/adapter screening subsection above, a biologist familiar with a project's vector, sequencing primers, and cloning chemistry enters into the database the two values required to determine this parameter. It not only facilitates accurate identification of vector and adapter fragments, but also permits their characterization as either VF1 or VF2, even though only one vector fragment might be present and even though the vector fragment might be masked by low quality base calls or other problems. Expected Vector Length also facilitates, in the absence of recognizable vector sequence, accurate identification of the expected adapter, of concatenated adapters that occasionally appear as a consequence of the cloning chemistry, and of a valid poly-T tail in a 3' EST. We have found these features to be distinct improvements, especially for single-pass sequencing projects such as ESTs.

To evaluate MAGIC-SPP with respect to adapter detection, we compared its performance to that of Lucy. A cDNA library with a relatively high frequency of adapter concatenation was selected for this purpose. The adapter was 11 nucleotides long. A total of 17,952 5' EST sequences produced by the Whitehead Institute were trimmed by both Lucy and MAGIC-SPP, using default parameters for comparison. The results are summarized in Table 1. In the case of Lucy, 16.6% of the passing sequence reads retained one or more adapter sequences after trimming, while with MAGIC-SPP only 4.4%, or 681 sequence reads, did the same. A text editor was used to estimate how many of these 681 sequence reads might have retained one or more adapter sequences because the templates from which they were derived contained three or more concatenated adapters. The text editor, which identified only perfect matches and which counted each input sequence only once regardless of the number of adapters, revealed that 673 of the 17,952 input sequences had a string of three or more. By extrapolation, 585 of the 15,596 passing sequence reads after trimming with MAGIC-SPP would be expected to have had three or more adapter sequences. Because the text editor requires a perfect match, it likely underestimates the number of input sequences with three or more adapters concatenated, since there might be an occasional base call error. Thus, since MAGIC-SPP looks only for the first two adapters, then it appears that MAGIC-SPP has missed fewer than 681 – 585 = 96. An obvious future goal thus becomes the conversion of this second adapter screen to an object-oriented module such that it can be used to detect concatenated adapters regardless of their length.

The Gap-Join/Seq-Merge algorithm introduced here is another novel feature that again has special value in single-pass sequencing projects. This algorithm is designed to deal in a more resourceful manner with the common problem of short low-quality regions, which are often found in DNA sequences and are referred to here as gaps. Commonly, high quality regions are identified by moving windows comparable to that used here. Lucy, for example, when faced with two or more high quality regions separated by one or more gaps selects the longest high quality region [2]. The processing pipeline for MAGIC-SPP, however, uses two additional moving windows to identify short and long gaps, placing information about those gaps in the database. By means of a fourth moving window (Fig. 4), Gap-Join/Seq-Merge then evaluates these gaps in sequential fashion with reference to the length of each following region of high quality. If the following region is sufficiently long, then the gap is merged into a growing region of high quality. If not, then the next gap together with the intervening region of high quality is added to the gap. The result is an intelligent inclusion of gaps when appropriate, and termination of the high quality portion of a sequence read when there is relatively little remaining to be saved by jumping over a gap. These longer DNA sequence reads are especially beneficial for downstream processing, in particular for EST clustering and annotation. Because information about the gaps is stored in the database, together with phred quality scores for every base call, this downstream processing can utilize that information to avoid potential difficulties.

Two examples are provided to illustrate how Lucy [2] and MAGIC-SPP, both using default parameters, differ with respect to deciding where to trim a sequence read. Figure 5 displays both base calls and phred quality scores [19] for a sequence read that is part of a demonstration data set for Lucy [27]. The region highlighted in green is the final high quality region identified by Lucy. The gap that terminates this region is centered at nucleotide 377; it consists of three base calls with a score of zero. The average error probability for Lucy's default _window_size2 of 10 is thus greater than the default maximum of 0.3, resulting in a division of the original high quality region into two segments. The shorter region is then discarded. In contrast, while Gap-Join/Seq-Merge would also identify this gap, it would nonetheless merge the two high quality segments into one longer read because of the additional 192 high quality base calls. Conversely, Figure 6 similarly displays a sequence read produced with MAGIC-SPP. In this example, MAGIC-SPP has chosen to jump over a short gap centered at nucleotide 588, thereby adding an additional 102 high quality base calls to the final high quality read. In contrast, Lucy would determine that the average error probability for the default _window_size2 of 10 is 0.402, well over the maximum permissible default value of 0.3. Consequently, Lucy would choose by default to divide the high quality region into two segments, choosing the longer as its final output. With Lucy, it is of course possible to obtain longer sequence reads by increasing the value for maximum average error probability. Doing so, however, will simultaneously increase the probability of including low quality fragments at the end of a sequence read because, unlike MAGIC-SPP, Lucy does not consider the length of any following high quality region when deciding whether or not to terminate a high quality region.

The results presented in Table 1 provide further evidence of the overall improvement that can be obtained with MAGIC-SPP as compared to Lucy, at least for a data set that is less than optimal. The yield of passing sequences is 86.9% with MAGIC-SPP as compared to 80.7% for Lucy, while MAGIC-SPP and Lucy gave average trimmed sequence lengths of 570 and 574 nucleotides, respectively. Consequently, MAGIC-SPP and Lucy provided total sequence of 8.89 and 8.33 × 10^6 ^nucleotides, respectively. Since 98.0% of all base calls in the sequence reads trimmed by MAGIC-SPP were equal to or greater than phred quality 20, it is evident that the overall sequence reads are still of high quality. This increase in yield is especially important for single-pass sequencing. In the case of ESTs, for example, primary objectives include facilitating gene discovery and developing gene models to assist with genome annotation. When given a choice of which of two high quality segments to retain, it might sometimes be preferable to retain the shorter because of its position in the sequence read. Moreover, discarding any substantial high quality region will have negative impact on achieving the two objectives noted here. Consequently, we suggest it is better to record gap information while retaining as many high quality base calls as possible, without significantly compromising overall sequence read quality. For those who wish to do so, it is of course possible to change the parameters of Gap-Join/Seq-Merge to better support other objectives.

Future goals are intended to address broader issues. While MAGIC SEQ-LIMS deals with a relatively generic, low-cost alkaline lysis miniprep, it will be useful to make it more versatile. We also plan to adapt MAGIC to one or more open-source relational database management systems such as MySQL [28] or PostgreSQL [29] so that the expense of Oracle can be avoided when that is an issue. Similarly, creating a simpler stand-alone system that uses configuration files in place of a relational database will make it useful to those who do not have access to the expertise needed to administer a relational database management system.

## Conclusion

MAGIC-SPP has been designed to minimize human error in a variable and demanding sequence processing environment, while simultaneously offering versatility, flexibility and automation. Its extensive interaction with a relational database, coupled with the unique combination of features introduced here, makes MAGIC-SPP a flexible and robust sequence processing package well suited to individual research laboratories and core facilities. Presently, about 500,000 sequence reads produced with MAGIC-SPP can be explored at and downloaded from a publicly accessible implementation of MAGIC DB using MAGIC SeqView [16].

## Availability and requirements

**Project name**: MAGIC-SPP

**Project home page**: 

**Project e-mail**: MAGIC-SPP@plantbio.uga.edu

**Operating system**: RedHat Linux 7.1 above

**Programming language**: Perl 5.8.0 and above, Java, JSP

**Other requirements**: Oracle 9i (9.2.0.6), Sun's J2SE version 1.4.2_06 or above, Tomcat 4.31, bioperl 1.4, phred, phrap, SSAHA

**License**: GNU GPL

## Authors' contributions

CL and M-MC-P designed the database schema, as well as the entire MAGIC system. CL and FS implemented Perl processing pipelines. CL, FS and HW implemented JSP interfaces. HW implemented Java GUIs. JQ contributed to database optimization and manuscript revision. JQ, CL and FS developed the UML representation. RMF compared MAGIC-SPP to Lucy. LHP and M-MC-P managed project development.

## Supplementary Material

Additional File 1 Supplementary figures for "MAGIC-SPP: a database-driven DNA sequence processing package with associated management tools" Click here for file

## Figures and Tables

**Table 1:** Comparative analysis of sequence trimming by Lucy and MAGIC-SPP. Both Lucy and MAGIC-SPP were used with their default parameters. The adapter for this cDNA library had a length of 11 nucleotides. All sequences are 5' ESTs.

	Lucy	MAGIC-SPP
Input sequences	17952	17952
Passing sequence reads	14490	15596
Sequence reads with at least one adapter remaining	2399	681
Average trimmed length in nucleotides	574	570
Total base calls in trimmed reads	8317260	8889720
