# Supplementary material for: MAGIC-SPP: a database-driven DNA sequence processing package with associated management tools
Source: BMC Bioinformatics. 2006 Mar 7;7:115. doi: 10.1186/1471-2105-7-115 (PMC1421442; doi:10.1186/1471-2105-7-115)
Supplement: Additional File 1 — Supplementary figures for "MAGIC-SPP: a database-driven DNA sequence processing package with associated management tools" [file 1471-2105-7-115-S1.pdf]

Supplementary Material

MAGIC-SPP: a database-driven DNA sequence processing package with associated management tools

Chun Liang[1], Feng Sun[1], Haiming Wang[1], Junfeng Qu[2], Robert M. Freeman, Jr.[3], Lee H. Pratt[1] and Marie-Michèle Cordonnier-Pratt[1]

[1]Laboratory for Genomics and Bioinformatics
Department of Plant Biology
University of Georgia
Athens, GA 30602, USA

[2]Department of Computer Science
University of Georgia
Athens, GA 30602, USA

[3]Department of Systems Biology
Harvard Medical School
Boston, MA 02115

14 February 2006

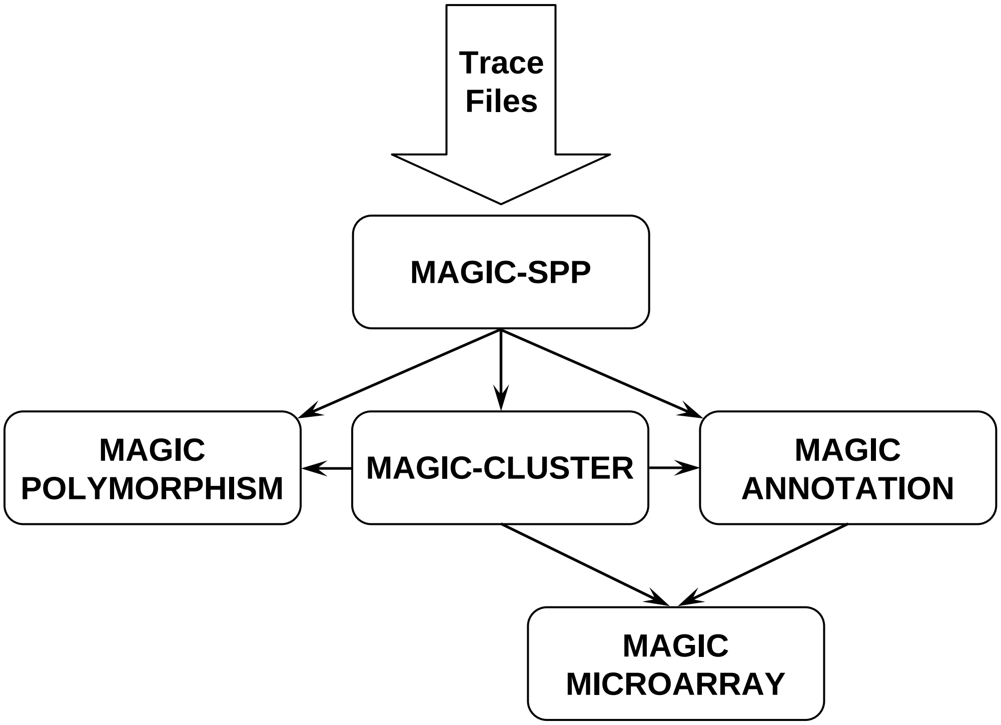

Figure 1: Relationship between MAGIC-SPP and other components of the complete MAGIC system. Information about sequence reads obtained from trace files processed through MAGIC-SPP is stored in MAGIC DB. While MAGIC-SPP can be used as a stand-alone package, it also provides input for four other major subsystems: annotation, EST clustering, detection of sequence polymorphisms, and MIAME-compliant microarrays. These downstream subsystems have been designed to benefit from features unique to MAGIC-SPP, for example the inclusion of genotype information in sequence names.

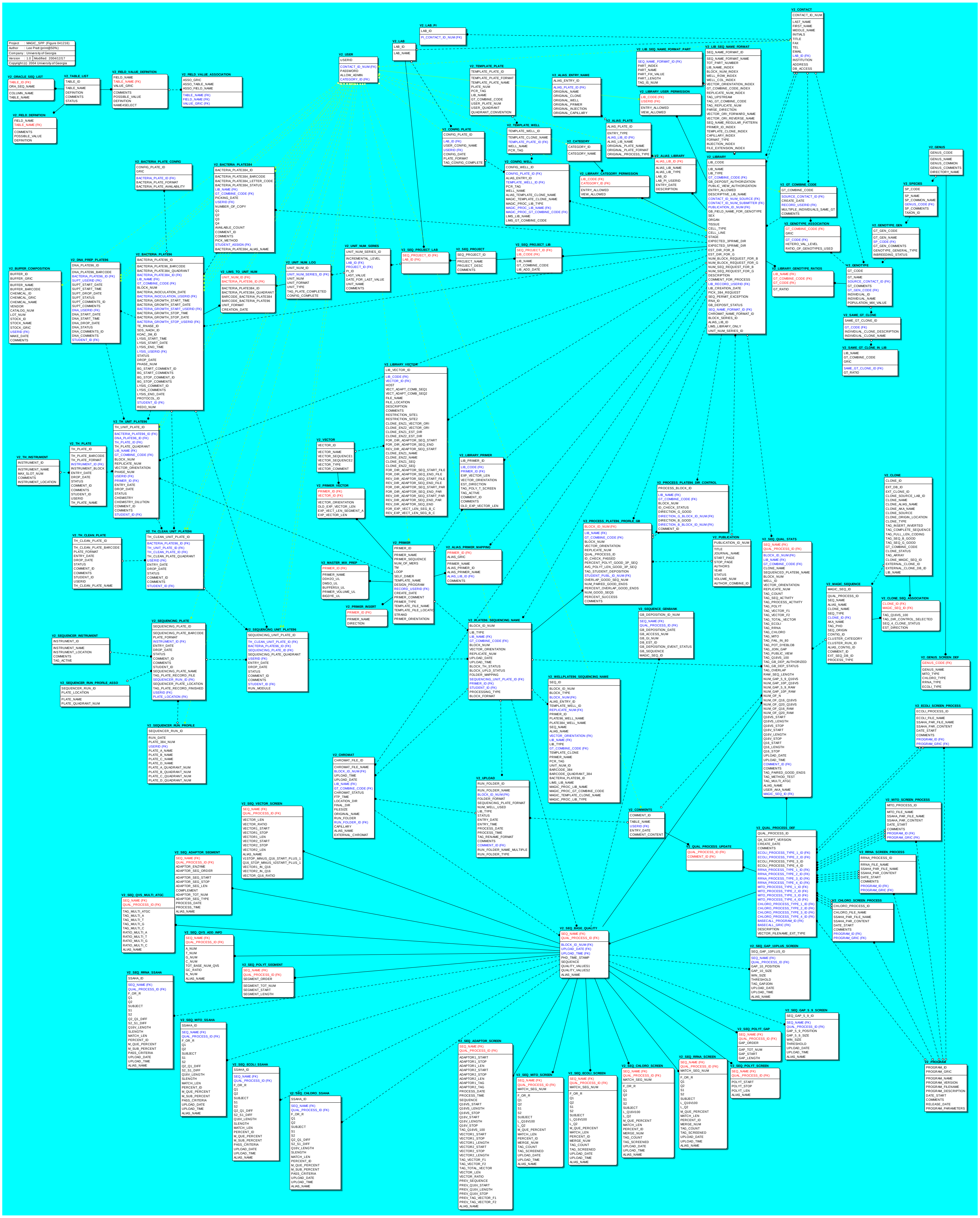

Figure 2: Entity-Relationship diagram of the MAGIC-SPP database. To see detail, use the ‘Zoom To…’ function in the Adobe Acrobat View menu.

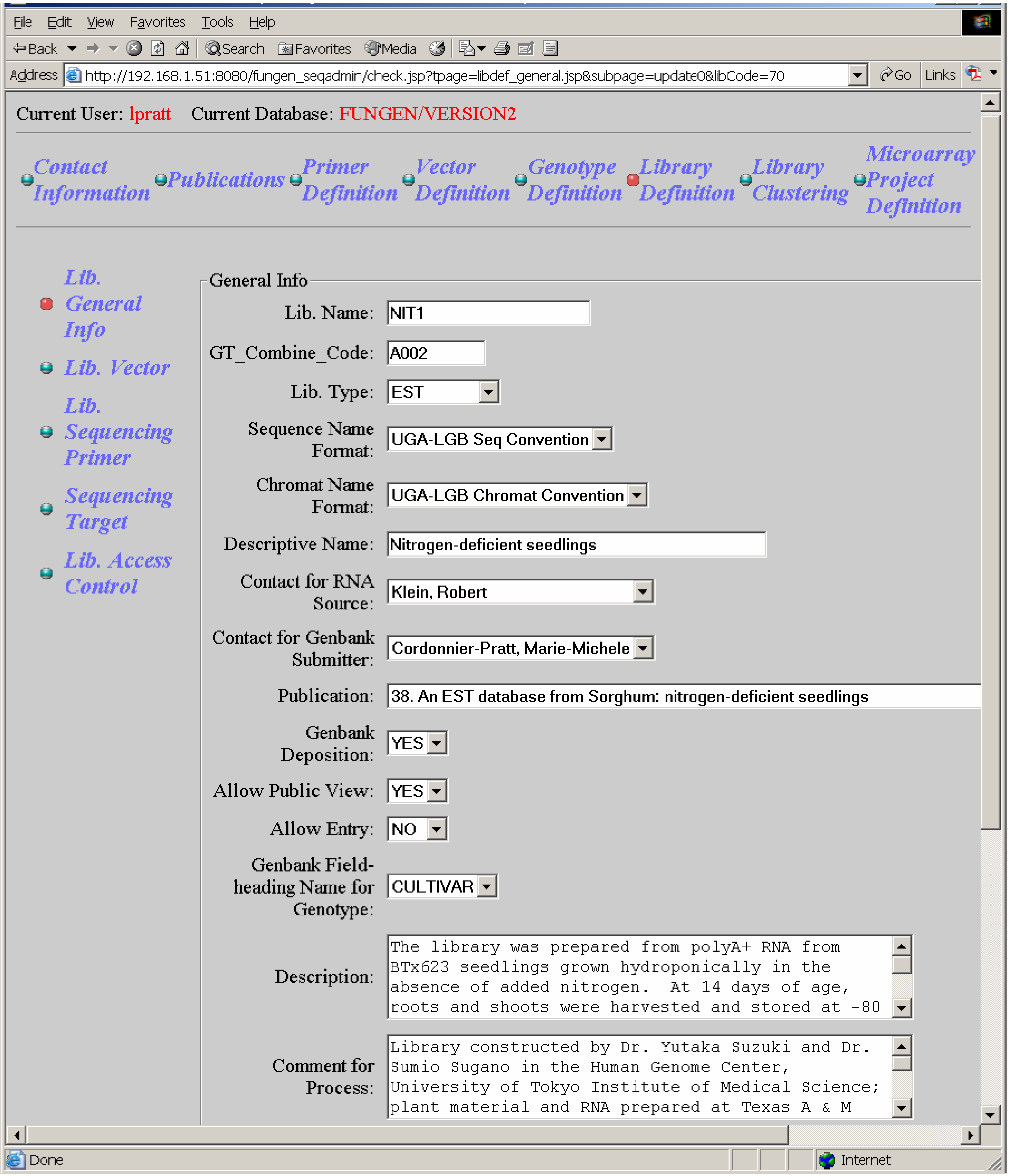

Figure 3: A screen shot of MAGIC Admin**.** In addition to one of the library definition screens illustrated here, note the many pages available not only for entry of contact and publication information, but also primer, vector and genotype definitions. In addition to what is shown, the sequencing primers and vector used with this library, together with all required information as described in the text, are entered using pages selected from the options along the left-hand border. These pages also permit input of target information for each sequencing project and specify user permissions with respect to both entry and viewing of data. The Contact Information page facilitates among other functions creation of user accounts.

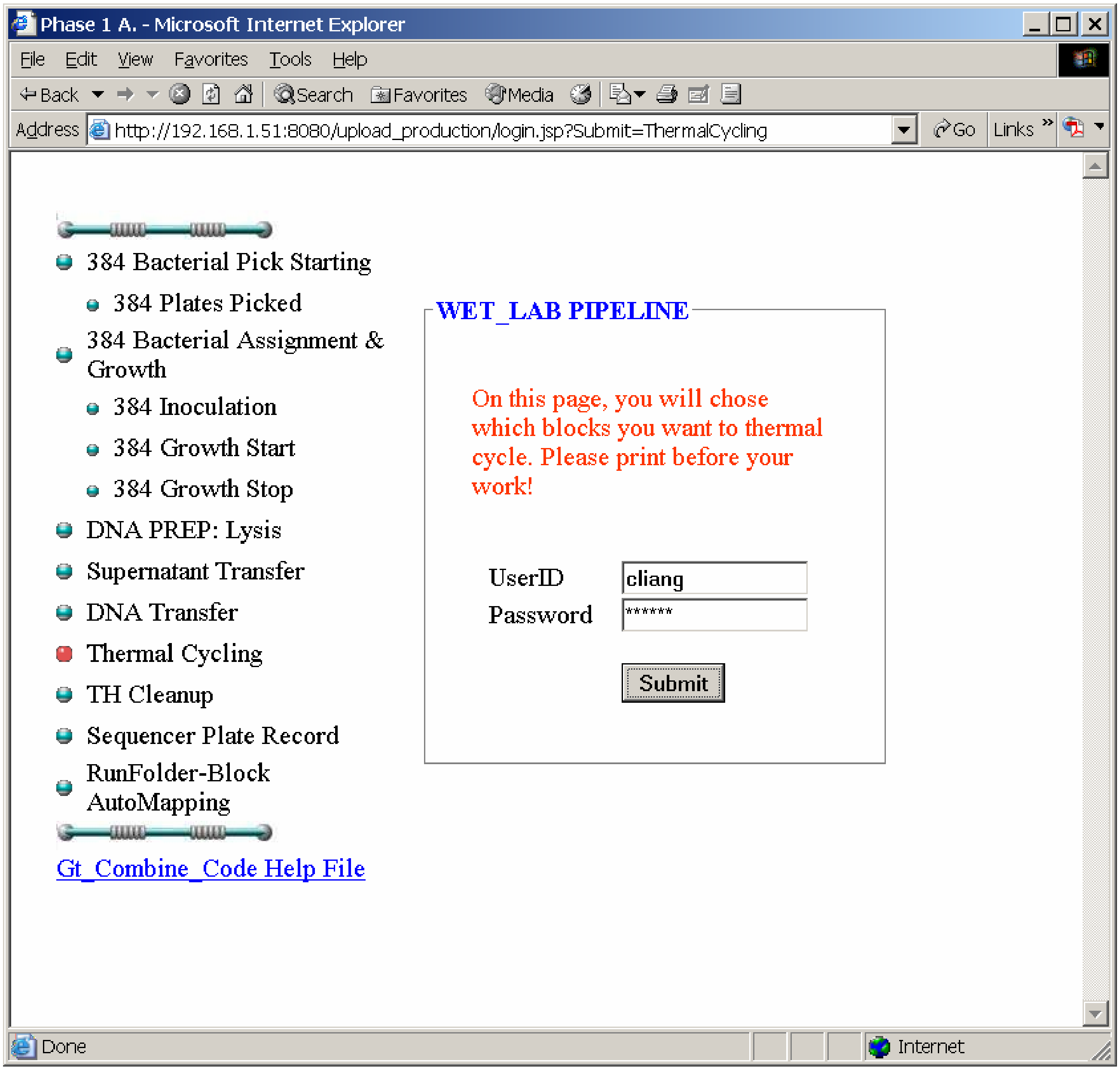

Figure 4: A screen shot of MAGIC SEQ-LIMS. This interface tracks all steps in DNA sequencing from picking of bacterial colonies to upload of trace files into the analysis server.

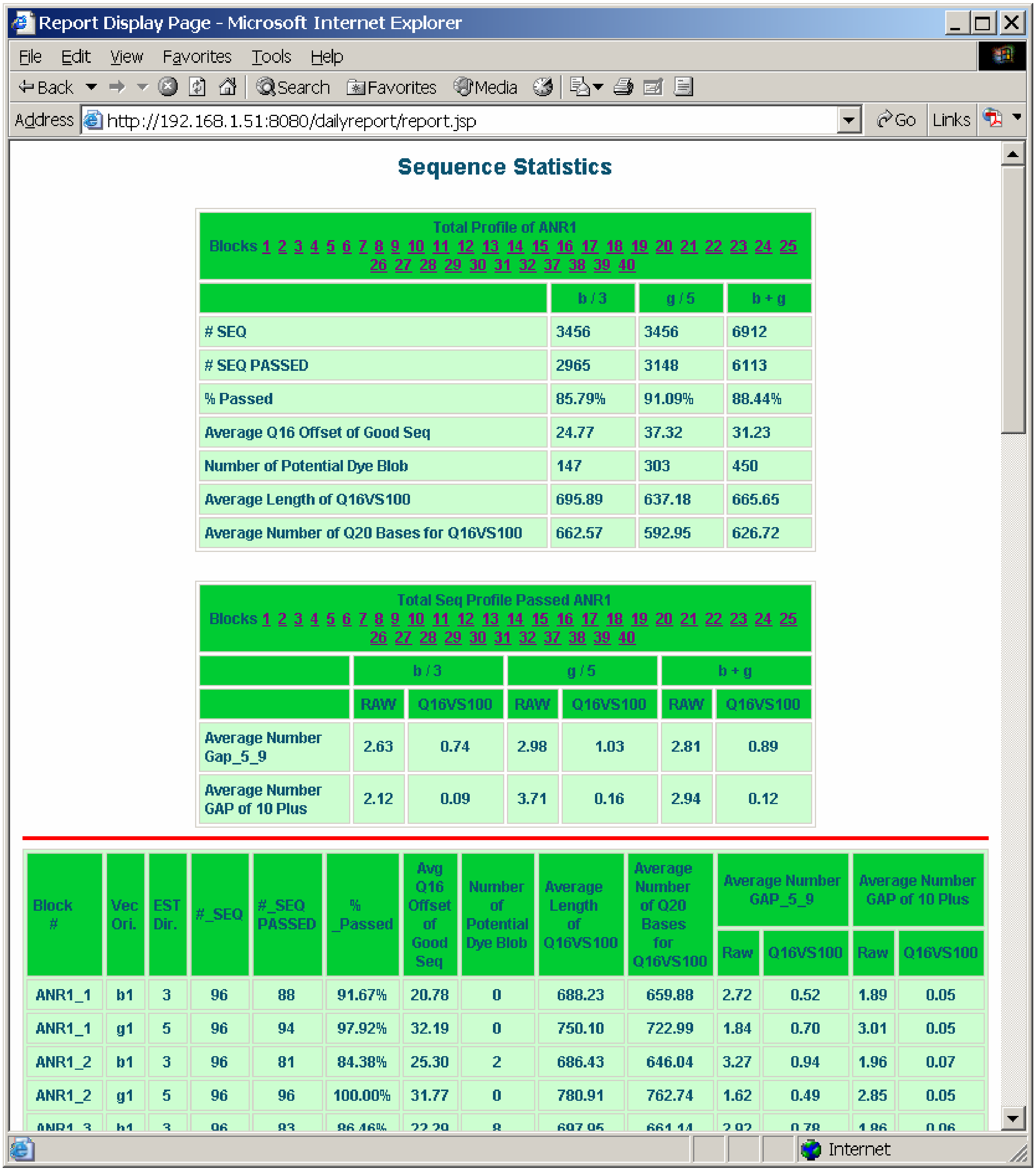

Sequence Statistics

Total Profile of ANR1
Blocks 1 2 3 4 5 6 7 8 9 10 11 12 13 14 15 16 17 18 19 20 21 22 23 24 25 26 27 28 29 30 31 32 37 38 39 40

	b / 3	g / 5	b + g
# SEQ	3456	3456	6912
# SEQ PASSED	2965	3148	6113
% Passed	85.79%	91.09%	88.44%
Average Q16 Offset of Good Seq	24.77	37.32	31.23
Number of Potential Dye Blob	147	303	450
Average Length of Q16VS100	695.89	637.18	665.65
Average Number of Q20 Bases for Q16VS100	662.57	592.95	626.72

Total Seq Profile Passed ANR1
Blocks 1 2 3 4 5 6 7 8 9 10 11 12 13 14 15 16 17 18 19 20 21 22 23 24 25 26 27 28 29 30 31 32 37 38 39 40

	b / 3		g / 5		b + g	
	RAW	Q16VS100	RAW	Q16VS100	RAW	Q16VS100
Average Number Gap_5_9	2.63	0.74	2.98	1.03	2.81	0.89
Average Number GAP of 10 Plus	2.12	0.09	3.71	0.16	2.94	0.12

Block #	Vec Ori.	EST Dir.	#_SEQ	#_SEQ PASSED	% _Passed	Avg Q16 Offset of Good Seq	Number of Potential Dye Blob	Average Length of Q16VS100	Average Number of Q20 Bases for Q16VS100	Average Number GAP_5_9		Average Number GAP of 10 Plus	
										Raw	Q16VS100	Raw	Q16VS100
ANR1_1	b1	3	96	88	91.67%	20.78	0	688.23	659.88	2.72	0.52	1.89	0.05
ANR1_1	g1	5	96	94	97.92%	32.19	0	750.10	722.99	1.84	0.70	3.01	0.05
ANR1_2	b1	3	96	81	84.38%	25.30	2	686.43	646.04	3.27	0.94	1.96	0.07
ANR1_2	g1	5	96	96	100.00%	31.77	0	780.91	762.74	1.62	0.49	2.85	0.05
ANR1_3	b1	3	96	83	86.46%	22.29	8	697.95	661.14	2.92	0.78	1.86	0.06

Figure 5: A screen shot of the MAGIC-SPP Sequence Statistics interface. This interface provides sequencing performance statistics for one or more plates in a selected library. In this view, all plates were selected for view both collectively (above red line) and individually (below red line).

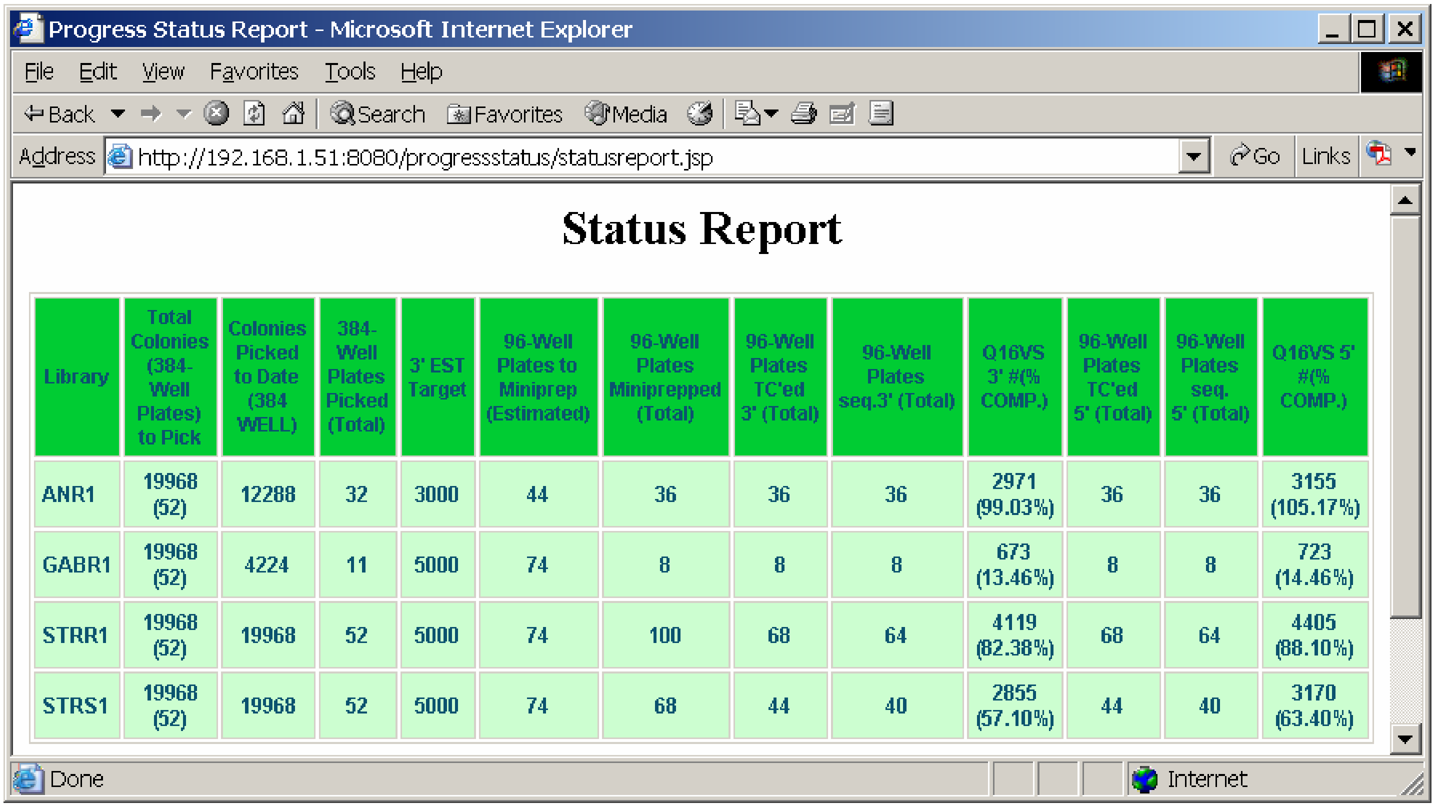

Status Report

Library	Total Colonies (384-Well Plates) to Pick	Colonies Picked to Date (384 WELL)	384-Well Plates Picked (Total)	3' EST Target	96-Well Plates to Miniprep (Estimated)	96-Well Plates Miniprepped (Total)	96-Well Plates TC'ed 3' (Total)	96-Well Plates seq.3' (Total)	Q16VS 3' #(% COMP.)	96-Well Plates TC'ed 5' (Total)	96-Well Plates seq. 5' (Total)	Q16VS 5' #(% COMP.)
ANR1	19968 (52)	12288	32	3000	44	36	36	36	2971 (99.03%)	36	36	3155 (105.17%)
GABR1	19968 (52)	4224	11	5000	74	8	8	8	673 (13.46%)	8	8	723 (14.46%)
STRR1	19968 (52)	19968	52	5000	74	100	68	64	4119 (82.38%)	68	64	4405 (88.10%)
STRS1	19968 (52)	19968	52	5000	74	68	44	40	2855 (57.10%)	44	40	3170 (63.40%)

Figure 6: A screen shot of the MAGIC-SPP Status Report interface. Target information, including number of colonies to be picked and number of sequences to be obtained, is entered through MAGIC Admin (columns 1, 2, 5 and 6 from the left). Other columns report progress. The query page for this interface also permits retrieving and viewing only data entered between any two dates.

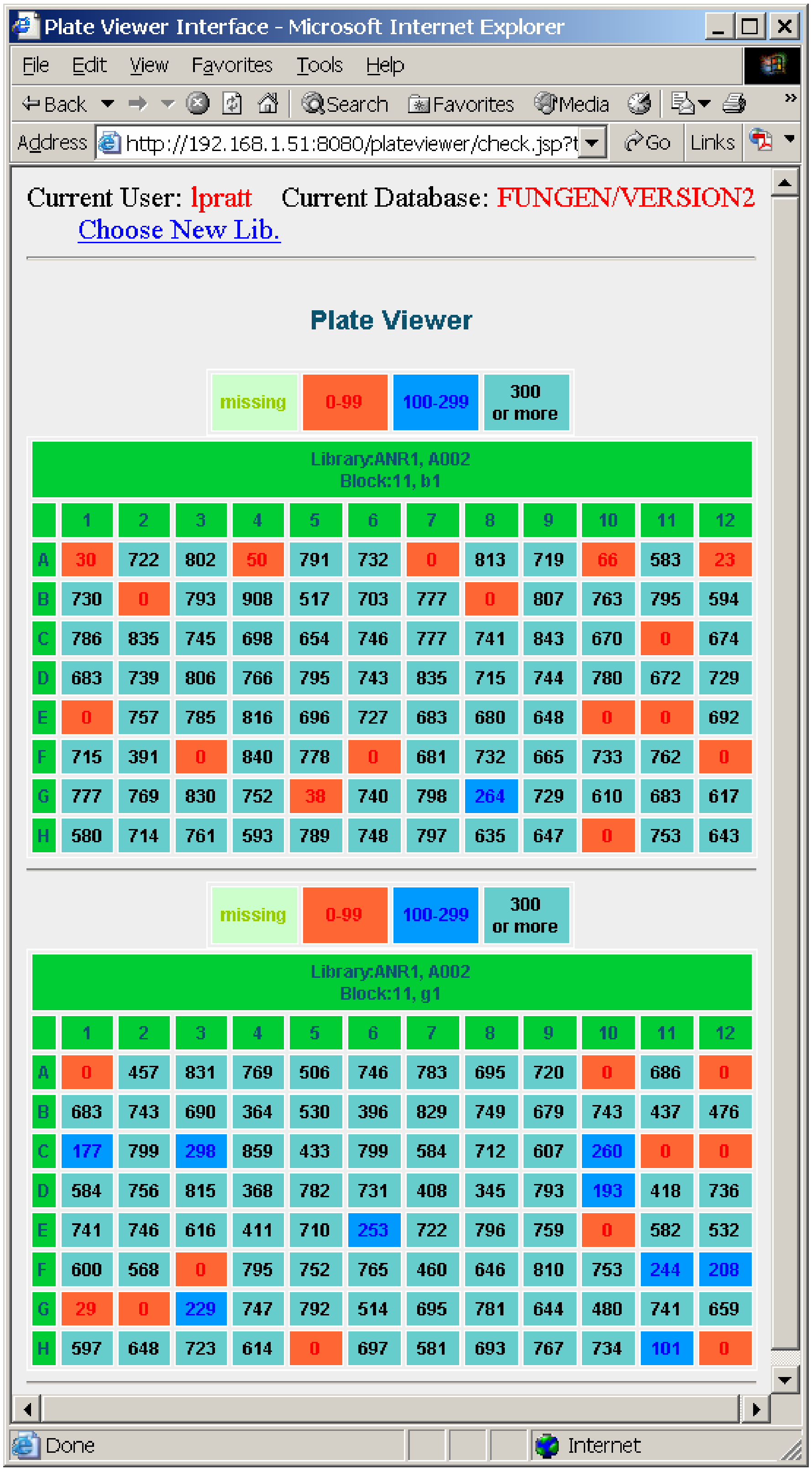

	1	2	3	4	5	6	7	8	9	10	11	12
A	30	722	802	50	791	732	0	813	719	66	583	23
B	730	0	793	908	517	703	777	0	807	763	795	594
C	786	835	745	698	654	746	777	741	843	670	0	674
D	683	739	806	766	795	743	835	715	744	780	672	729
E	0	757	785	816	696	727	683	680	648	0	0	692
F	715	391	0	840	778	0	681	732	665	733	762	0
G	777	769	830	752	38	740	798	264	729	610	683	617
H	580	714	761	593	789	748	797	635	647	0	753	643

	1	2	3	4	5	6	7	8	9	10	11	12
A	0	457	831	769	506	746	783	695	720	0	686	0
B	683	743	690	364	530	396	829	749	679	743	437	476
C	177	799	298	859	433	799	584	712	607	260	0	0
D	584	756	815	368	782	731	408	345	793	193	418	736
E	741	746	616	411	710	253	722	796	759	0	582	532
F	600	568	0	795	752	765	460	646	810	753	244	208
G	29	0	229	747	792	514	695	781	644	480	741	659
H	597	648	723	614	0	697	581	693	767	734	101	0

Figure 7: A screen shot of MAGIC-SPP Plate Viewer. This interface assists with troubleshooting by showing sequences from both vector directions for the same plate. For example, in this illustration wells A1, A10, A12, C11, and E10 have all failed in both forward (top) and reverse (bottom) sequencing directions indicating that these failures are most likely due to missing or poor quality template DNA. Numbers within wells represent Q16VS read lengths.
